# Studying The Effect of Infertility on Marital Violence in Turkish Women

**Published:** 2013-03-03

**Authors:** Aygul Akyuz, Memnun Seven, Gonul Şahiner, Bakır Bilal

**Affiliations:** 1 Department of Obstetrics and Gynecologic Nursing, Gulhane Military Medical Academy, School of Nursing, Ankara, Turkey; 2 Department of Public Health, Gulhane Military Medical Academy, School of Medicine, Ankara, Turkey

**Keywords:** Infertility, ,, Violence, Risk Factor

## Abstract

**Background:**

The aim of this descriptive study was to evaluate the level of marital violence among Turkish women and to determine whether infertility was a risk factor for marital violence.

**Materials and Methods:**

This descriptive study was conducted during January-July 2009 at a training hospital. The study groups comprised 204 fertile and 228 infertile women. We administered the Descriptive Information Questionnaire and Scale for Marital Violence against Women (SDVW) to obtain data.

**Results:**

There was a statistically significant difference between infertile and fertile women for the total score of violence in marriage. The emotional, economic and sexual violence scores were higher in the infertile group. However, the verbal violence score was lower

**Conclusion:**

We performed a detailed study aimed at uncovering the presence of any violence from the data collection stage to the end of treatment in infertile couples with the intent to include questions to this effect in the care plan.

## Introduction

Violence is an important community health problem commonly observed among all cultures worldwide regardless of geographical boundaries, economic development and educational level (1- 4). The most common type frequently hidden is violence toward women (1). Violence directed at women is defined as any behavior including those which can cause physical, sexual or psychological harm, or cause pain, as well as the threat of such behavior and restriction of women's freedom by force. Violence against women is most often experienced within the family. According to a 2005 report by the World Health Organization (WHO), the proportion of women who have suffered physical violence by a male partner was 13-61% (1, 2, 4).

Numerous studies have emphasized the importance of violence-related factors that trigger the initiation of marital violence (1-3, 5). The WHO reported the following common factors among women who experienced violence (1). Individual factors included the woman’s level of education, employment status, previous victimization, level of social support, and a history of violence in her family. Partner factors included the level of communication with the woman, substance abuse such as alcohol and drugs, employment status, whether he had witnessed violence between his parents during childhood, and whether he was physically aggressive towards other men. Social structural factors included the level of economic inequality between men and women; women’s independence; attitudes towards gender roles and violence; and whether family, neighbours, and friends partially intervened in domestic violence (1-3, 5, 6). Consequently, violence cannot be attributed to a single cause.

The combination of causes negatively influence the couple's psychosocial and physical well-being (7). It leads to occur a marital crisis. The negative effects they produce over the short or long term can lead to violent behavior if the couple cannot cope with these stressors and do not have adequate social support (7-9).

Childbearing is an important goal for couples. Thus, infertility is one of the most important causes for a crisis, as it negatively affects a couple's relationship (10-12). Following an infertility diagnosis, the couple begins to have physical, psychological and financial problems. They experience a stressful period that often encompasses their sexual life. The stressful period is created by the high cost of treatment procedures and costly tests; ongoing visits to physicians and infertility clinics that are sometimes located in distant cities which require extensive traveling; and abiding by a definite sexual intercourse timetable established by the physician. The pressures of family and society to have a baby as soon as possible and not being able to explain the problem are additional causes for stress and conflicting emotions (10, 11, 13-16).

Wang et al. have reported that the diagnosis and treatment of infertility significantly decreases marital quality, by having a negative effect on the couple's sexual life, their communication with family and friends, role distribution in the family, and conflict resolution (17). These factors that decrease marital quality and satisfaction are also reported to be risk factors that contribute to marital violence (7-10,17). However, only a few studies have been published regarding the relationship between infertility and marital violence. Leung et al. have reported a 1.8% prevalence of lifelong marital violence for women who underwent treatment for infertility (18). Ameh et al. have reported that 41.6% of infertile women experience marital violence as a result of infertility. These women have a higher risk of being the recipients of marital violence than fertile women (19). Yıldızhan et al. report that 33.6% of infertile women are exposed to marital violence and that 78% experience marital violence after the diagnosis of infertility (20). Similarly, in a report by Ardabily et al. (12) 61.8% of infertile women experience marital violence. The violence against infertile women, although considerable, is an unreported problem. We therefore believe studying the infertility-violence relationship in different societies and in couples from different socioeconomic levels will increase the reliability of the findings related to marital violence against infertile women.

The aim of this descriptive study was to determine the level of marital violence in a group of Turkish women and establish if infertility was a risk factor for experiencing marital violence. The evaluation was achieved by considering the following questions. Is there a difference between infertile and fertile women regarding the level of marital violence? Is there a difference between infertile and fertile women regarding the type of marital violence? What are the risk factors responsible for marital violence in infertile Turkish women?

## Materials and Methods

### Participants

This descriptive study was conducted during January-July 2009 at a training hospital in Ankara, Turkey. The women included in the study were separated into two groups, infertile and fertile. The fertile and infertile group were not matched with respect to certain variables; all eligible women who met the criteria for participation were included.

The infertile group (n=228) consisted of women who referred to the Infertility Center of the training hospital during the study period, who were treated for primary infertility because of male, female or unexplained problems. A total of 300 women in the infertile group were seen at the Infertility Center during the seven-month data collection period; 260 who met the criteria for participation were asked to participate in the study. Of those selected, 234 (90%) consented to participate. However, 6 out of the 234 women had incomplete data, therefore the final sample comprised 228 (87%) in the infertile group.

The fertile group (n=204) consisted of married women with children and no history of fertility problems who attended the Gynecology Outpatient Department of the same hospital during the same period. A total of 1000 fertile women visited the Gynecology Outpatient Department during the data collection period; 400 met the criteria for participation and were selected to participate in the study. Of those selected, 355 (89%) consented to participate. However, 125 of the 355 consenting women had incomplete data, thus we reduced the final sample to 204 (51%) in the fertile group.

### Instrument

We administered the Descriptive Information Questionnaire and Scale for Marital Violence against Women (SDVW) for data collection.

The SDVW was developed by Betül Kılıç in 1999 for Turkish women to determine the level of marital violence. The alpha values obtained during the development of the scale ranged from 0.73 to 0.94. The SDVW is a 50-item self-reported scale, wherein each item consists of three statements in five subscales of violence: physical, emotional, verbal, economic, and sexual. Numerical values from 1 to 3 are assigned to each item in the marked statements. The total violence scores are obtained by adding these points and range from 50 to 150. The total score demonstrates the level of marital violence experienced by the woman. There is no specific cutoff score in this scale to determine women who have experienced marital violence (21). Cronbach’s alpha coefficient was calculated as 0.78 in this study.

The Descriptive Information Questionnaire was developed by the present investigators after evaluation of the relevant literature. Obstetrics experts examined the questionnaire's content validity to confirm general appropriateness and applicability. The questionnaire consisted of 12 socio-demographic questions that included the ages of the women and their spouses, level of education, occupational status, age at first marriage, and infertility characteristics. The prepared questionnaire was first administered to 20 fertile women at the Gynecology Outpatient Department of the hospital, as a pilot study to ascertain whether the items could be easily understood.

### Procedure

The hospital Institutional Review Board approved this study. Participating women provided verbal consent after the aim and method of the study were explained. The investigator completed survey forms through faceto- face interviews with each woman. The average time for an interview was approximately 20 minutes.

### Data analysis

The SPSS 11.0 software package was used for statistical analysis. The distribution of the data was expressed as counts and percentages. Chi-square, Kruskal-Wallis, and t tests were used for statistical comparison between groups, as appropriate. The Kruskal-Wallis test compared variables that were not normally distributed. Linear regression analysis was used to explore the relationship between Predicted variables on the levels of marital violence. We chose the backward method for linear regression analysis. Descriptive statistics were presented using the arithmetic mean and standard deviation. A p value of less than 0.05 was accepted as statistically significant.

## Results

Totally, there were 228 infertile and 204 fertile women included in the study. The mean age of women was 29.54 ± 4.268 years in the infertile group and 30.40 ± 4.907 years in the fertile group. Table 1 presents the descriptive features of the women in fertile and infertile groups.

Table 2 presents the infertility characteristics of women in the infertile group of which 37.7% were diagnosed with unexplained infertility. A total of 28.1% underwent infertility treatment for three or more years.

Table 3 presents the mean scores from both groups of women for the SDVW total and violence subscales. The mean total violence score of women was 67.23 ± 8.037 in the infertile group and 64.49 ± 5.166 in the fertile group, according to the SDVW. There was a statistically significant difference between the infertile and fertile women in the total violence score and mean scores of emotional, economic and sexual violence. Although the infertile group had higher emotional, economic and sexual violence scores, however their verbal violence score was lower. There was no statistically significant difference between the groups for the physical violence score.

**Table 1:** Socio-demographic features of the study participants


	Infertile group n=228		Fertile group n=204	
	n	%	n	%

Age (Y)				
**<30**	127	55.7	107	52.5
**≥30**	101	44.3	97	47.5
**Mean ± SD**	29.54 ± 4.268		30.40 ± 4.907	
**Age at first marriage**	22.76 ± 3.427		22.22 ± 3.235	
Educational status				
**Primary school**	58	25.4	68	33.3
**High school or above**	170	74.6	136	44.4
****				
Employment status				
**Not working**	183	80.3	148	72.5
**Working**	45	19.7	56	27.5
Duration of marriage (Y)				
**0-5**	120	52.6	39	19.1
**6-10**	88	38.6	90	44.1
**10 or higher**	20	8.8	75	36.8


**Table 2:** The infertility characteristics of infertile women


n=228	n	%

Infertility cause		
**Male-related problems**	69	30.3
**Female-related problems**	38	16.7
**Male and female related problems**	35	15.4
**Unexplained**	86	37.7
**Duration of infertility treatment (Y)**		
**≤2**	164	71.9
**≥3**	64	28.1


**Table 3:** Comparison of SDVW scores


	Infertile group n=228	Fertile group n=204		
	Mean ± SD	Mean ± SD	t	P value

**Total SDVW score**	67.23 ± 8.037	64.49 ± 5.166	4.157	0.000
**Violence subscale scores**				
**Physical**	10.46 ± 1.648	10.38 ± .652	0.599	0.550
**Emotional**	16.16 ± 1.956	15.02 ± 1.255	7.105	0.000
**Verbal**	13.71 ± 2.072	14.37 ± 2.115	2.970	0.003
**Economic**	14.37 ± 2.612	12.93 ± 1.818	6.562	0.000
**Sexual**	12.53 ± 2.200	11.84 ± 1.490	3.458	0.000


SDVW score. Education status, age at first marriage for couples, and couples' fertility status were included in the model as they had a statistically significant relationship with SDVW. This indicated that infertility was a contributing factor for experiencing marital violence when combined with other risk factors such as women’s' educational status and age at first marriage. The correlation between total SDVW score and variables obtained from the model was 0.385. The Durbin- Watson coefficient had a value of 1.739, which demonstrated that our model was well formed (Table 4).

**Table 4:** Results of regression analysis between the independent variables effecting total SDVW score


n=228	Total SDVW score		
	B	t	P value

**Independent variables**			
**Age at first marriage (Men)**	-0.312	-5.109	0.018
**Age at first marriage (Women)**	-0.267	-4.063	0.019
**Educational status of the women**	-1.249	-2.363	0.000
**Fertility status of couples**	-3.195	-2.378	0.000
	**R**	**R square**	**Durbin-Watson**
**SDVW model**	0.385	0.148	1.739


In the infertile group we observed a statistically significant relation between SDVW total score and infertility cause (p=0.023) and duration of infertility treatment (p=0.000). Infertile women with femalerelated or unexplained problems had a higher SDVW total score as did those who underwent infertility treatments for three or more years (Table 5).

**Table 5:** Comparison of the infertility features of couples in the infertile group and SDVW scores


n=228	SDVW total score		
	Mean ± SD	X^2^/t	P value

Infertility cause*		
**Male-related**	64.55 ± 6.057	9.575	0.023
**Female-related**	67.30 ± 9.162		
**Male- and female-related**	65.83 ± 7.036		
**Unexplained**	68.92 ± 7.916		
Duration of infertility treatment** (Y)			
**≤2**	66.14 ± 6.440	-3.344	0.000
**≥3**	70.02 ± 10.699		


*; Kruskal-Wallis test and ** ; t test.

## Discussion

We have presented data regarding the experience of violence from infertile and fertile women, the types of violence experienced, and comparisons regarding these characteristics. There are few studies of infertile women who have experienced violence. These studies have included only infertile women in their studied population, whereas the current study included both infertile and fertile women. We evaluated all types of violence, not just the general concept of violence. Thus, our study was unique.

We found the mean violence score of the infertile group to be higher than the fertile group. The few studies on this subject have also reported higher levels of marital violence experienced by infertile women compared to fertile women, which supported the current study (18-20). Ameh et al. have found that 41.6% of infertile women experience marital violence because of infertility and they have a higher risk of exposure to marital violence than fertile women (19). Yıldızhan et al. have found that 33.6% of infertile women are subjected to violence; 78% of these women experience violence for the first time after the diagnosis of infertility (20). Similarly, Ardabily et al. have demonstrated that 61.8% of infertile women experience marital violence because of their infertility (12). Pasi et al. (22) have also reported that 76.3% of infertile women and 65.9% of those who have at least one child have experienced violence. In their study there was a highly significant association between infertility and domestic violence.

Violence can be divided into economic, emotional, sexual and verbal in addition to physical groups (12, 23). We evaluated the women according to physical, emotional, sexual, economic, and verbal violence subtypes in our study. There was no difference between infertile and fertile women regarding physical violence. The 2005 WHO report has stated a worldwide rate of physical violence as high as 13-61% for women (1).

Some studies have estimated the physical violence rate to be 14-33% for infertile women (12, 18). Ameh et al. report a physical violence rate of 17.5% for infertile women, which they state is similar to the general population (19). In contrast, Pasi et al. (22) have reported that infertile women experienced physical or sexual violence more frequently than fertile women, although these researchers assessed physical and sexual violence together.

We found higher levels of sexual, economic and emotional violence among infertile women compared to fertile women in this study. Leung et al. also reported a rate of 55.6% for emotional violence in infertile women (18). Ardabily et al. (12) have reported a psychological violence rate of 33.8%, which makes it the most common type of violence. Ameh et al. (19) have found a psychological violence rate of 51.5% and economic violence rate of 6.2% in infertile women. Similarly, Yıldızhan et al. (20)have also reported rates of economic violence of 29.2% and sexual violence in the form of being forced to have sexual intercourse at a rate of 7.3% for infertile women. Pasi et al. have reported that approximately three quarters of either infertile or fertile women have experienced emotional violence. Although the rate of emotional violence in infertile women was higher than in fertile women, it was not statistically significant (22). Totally, these findings indicated that infertile women have been subjected to high rates of emotional, sexual and economic violence. Studies have shown that repeated unsuccessful infertility treatments deteriorated the marital relationship (10, 17, 24) and decreased sexual satisfaction (14, 25, 26), while the financial burden of the treatment increased marital conflict (14, 24). The high emotional, sexual and economic violence scores for infertile women in this study were possibly due to marital conflicts that resulted from infertility and its treatment.

We have found that infertility is a factor which contributes to marital violence when combined with other risk factors such as women’s' educational status and age at first marriage. In this study, infertile women with less education who married at a younger age were more likely to have experienced marital violence. Similarly, Akyüz et al. (27) have reported a negative correlation in women between educational level, age at first marriage and first sexual intercourse with the level of violence. Rickert et al. reported that women who suffered from violence had their first sexual intercourse at younger ages (28). Current literature has also confirmed that most women who experienced violence had lower educational levels (5, 9, 28). However some studies have shown that educational level did not effect the experience of violence of women (20). Violence is influenced by culture. Depending on cultural differences, among societies the rates of women who experience violence might differ as well as the factors that affect marital violence (9). Some socio-demographic factors such as the place of women in society, educational level, employment status and independent status of women might lead to the perception that infertility is only a woman's problem. Similarly, it is stated that violence aganist women occurs more often in a male-dominant social structure (27). Therefore, it is thought that infertile women with lower educational levels who marry at younger ages may be more affected by male dominance. These women may have less autonomy and be more dependent on their husbands. Similarly, Kocacık et al. have reported that the extent of male dominance is associated with domestic violence; a woman is less likely to be abused in households where decisions are made collectively. It has been reported that families where women have to obtain permission from the husband to perform some activities have a higher risk of experiencing violence from the husband (5).

Unexpectedly, we found a lower level of verbal violence experienced by infertile women compared to fertile women. Generally, it has been presumed that verbal violence among infertile women is possibly higher than fertile women. In support, other studies report that verbal violence is one of the common forms of violence experienced by infertile women. According to one study, most infertile women have stated that their husbands humiliated and insulted them because of their infertility (29). Ameh et al. have reported that 39.2% of women are subjected to verbal violence and 27.8% are subjected to such violence by being made fun of (19). Yıldızhan et al. have stated that verbal abuse is the most common type of domestic violence, which 63.4 % of infertile women report having experienced verbal violence, followed by ridicule and threats of violence. However, it has also been reported, that the results are not representative of the situation among infertile couples who have male factor infertility. Verbal violence is common among women with female factor infertility (20). It is therefore thought that differences among studies’ results can arise from methodological differences. In addition, both the infertility and violence experiences and the responses of couples differ from community to community and even within the same community (1, 4, 10). Infertile women are affected psychologically to a larger degree than men in the Turkish society, which makes infertile women more sensitive and emotional. It is possible that husbands attempt to act more carefully and choose their words. In general, it is considered that women are unable to conceive regardless of the real cause.

Women may suffer from a feeling of low self-esteem, guilt, and embarrassment about not being able to become pregnant. They may feel, on some level, that they deserve verbal violence and these feelings may cause them to ignore verbal violence. We believe this can be the reason for the low verbal violence scores.

In this study, the level of marital violence was higher in infertile women with unexplained and female-related problems. This result showed that infertile women with unexplained or female-related problems might be at increased risk for marital violence compared to those with male or male-female problems. It was stated that the prevalence of domestic violence against women with female factor infertility ranged between 33.6% and 61.8% (5, 12). It has been proposed that the socialization process promotes different meanings of fertility for men and women as a reflection of virility for the man, whereas motherhood is more central to the female identity in some societies (30). In general, it is believed that women are the cause for the infertility regardless of the actual cause, particularly in male-dominated societies. Women have often been punished psychologically, socially and economically based on the belief that they are the cause of the infertility. Furthermore, men have been shown to be likely to act violently when virility and the ability to continue their bloodline is in danger due to infertility, particularly when the wife is responsible (12, 18-20).

The level of marital violence was higher in those women who had longer durations of infertility treatment in this study. However, by contrast, some studies reported no significant relationships between domestic violence and infertility duration (5, 12, 19). It has been proposed that long-lasting infertility and unsuccessful treatment cycles intensify the stress that might lead to marital violence.

## Conclusion

In the study, we observed that infertile women were at an increased risk for marital violence compared to fertile women. The experience of physical violence was similar in both groups. However, the level of experiencing sexual, economic and emotional violence in infertile women was higher than fertile women. The level of verbal violence was lower among infertile women compared to fertile women. It was found that marrying at a young age and less education in infertile women were factors that affected marital violence. It was also found that infertile women with unexplained or female-related problems and longer duration of infertility treatment had increased risk for marital violence, compared to those with male or male-female problems.

The results of this study have demonstrated that infertile women are more likely to have experienced violence. The violence experienced by infertile women, who are already psychosocially negatively affected will lead to more destructive changes in these women. It is therefore necessary to carry out observations aimed at uncovering the presence of any violence from the data collection stage to the end of treatment in infertile couples and to include questions to this effect in the care plan. Both infertility and violence experience may be affected by social structure and culture. In the male dominant society, particularly women with female-related or unexplained problems can be under more pressure due to not being able to maintain family continuity. The role of maintaining family continuity by childbearing is considered the woman's primary role. Even in some societies such as Turkey, in which continuing bloodline is an important aim for a marriage, infertility can be a reason for divorce. Similarly, Yıldızhan et al. in a Turkish study, have stated that 87% of abused, infertile women were threatened with divorce by their husbands (20). Thus, women might have feelings of guilt, embarrassment and fear due to infertility. When subject to violence, women usually attempt to keep this a secret (31). Violence can be also evaluated as a secret as well as a problem that just involves family members. Therefore, it may be necessary to uncover and intervene in marital violence by taking into consideration characteristics of the society, before being subjected to violence is stated by infertile women as well as all women.

Healthcare staff must provide the necessary education and recommendations to emphasize that this behavior is unacceptable for those who experience violence.

### Limitations

There are a number of limitations inherent in this study, thus these findings cannot be generalized. Our study sample consists of a group of Turkish women conducted at a single center. The measurement instrument used for the study provides a violence score for the violence experienced and type of violence. However, it is not possible to use a classification that qualifies study subjects as experiencing/not experiencing violence. It therefore does not provide a percentage for women experiencing violence. Studies in the literature use various measurement instruments that evaluate the rates of experiencing violence and the type of violence. This makes it more difficult to compare the results of different studies.

The violence measurement instruments used vary among the few studies in the literature. There is no cut-off point for the violence measurement instruments used in this study. We suggest that other studies that compare the violence rate in infertile and fertile women to be performed with instruments that have a cut-off point.

Both violence and infertility can be influenced by sociocultural differences. We therefore strongly suggest that studies, similar to those already performed, determine the rate of being subjected to violence by infertile women be undertaken in groups with other sociocultural characteristics.
